# News and Events of the Week

**Published:** 1911-06-10

**Authors:** 


### NEWS AND EVENTS OF THE WEEK.

A Subscription list has been opened for the purpose of erecting a monument to the late Dr. Claude Martin, of Lyons, well known for his works on stomatology. M. Caillon, 1 rue Victor Hugo, Lyons, will be pleased to receive subscriptions.

Professor Metchnikoff, assistant director of the Pasteur Institute of Paris, has left for Russia to study in the Astrakan Province the bacilli of plague and the conditions under which they resist summer heat. The Mission consists of four members, two of whom are of Russian nationality.

The German Central Society for the care of infancy has decided to build a house for the pedagogic education of "psychopathic children." In the same way the Medical College of Prussia has resolved to organise a "whooping-cough colony" at the seaside. Houses for families are to be built near a forest, separated from each other by plantations.

The Second Biennial Congress of the Far Eastern International Association of Tropical Medicine is to be held in Hong-Kong from January 20 to 27. A scientific committee has been appointed to classify the papers submitted so as to give as far as possible a day to the following subjects: Protozoology and helminthology; cholera, plague, leprosy, and tuberculosis; tropical fevers, including malaria, beri-beri, and dysentery; surgery, obstetrics and infantile diseases; climate, hygiene, and sanitation.

On June 30 the season of the Orient Line pleasure cruises to Norway opens with the despatch of the s.s. *Otranto* for a fortnight's cruise to Norway, visiting also those interesting Baltic capitals, Copenhagen, Gothenburg, and Christiania. The *Otranto* is 12,124 tons, and one of the five large twin-screw vessels recently added to the Orient fleet. She has a very fine provision of exercise decks, and, besides numerous single-berth cabins, has bedstead state-rooms and cabines de luxe with private sitting-room and bathroom attached. There will be five cruises this year with varying programmes, and the fare ranges from twelve guineas upwards. An illustrated descriptive booklet will be sent on application to the Orient Company's Head Office, Fenchurch Avenue.

